# Incident Chronic Kidney Disease and Newly Developed Complications Related to Renal Dysfunction in an Elderly Population during 5 Years: A Community-Based Elderly Population Cohort Study

**DOI:** 10.1371/journal.pone.0084467

**Published:** 2013-12-18

**Authors:** Shin Young Ahn, Jiwon Ryu, Seon Ha Baek, Sejoong Kim, Ki Young Na, Ki Woong Kim, Dong-Wan Chae, Ho Jun Chin

**Affiliations:** 1 Department of Internal Medicine, Seoul National University Bundang Hopsital, Seong-Nam, Korea; 2 Department of Internal Mecidine, Seoul National University College of Medicine, Seoul, Korea; 3 Department of Neuropsychiatry, Seoul National University Bundang Hopsital, Seong-Nam, Korea; 4 Renal Institute, Clinical Research Center, Seoul National University College of Medicine, Seoul, Korea; University of São Paulo School of Medicine, Brazil

## Abstract

**Background:**

Few studies have evaluated the association between incident chronic kidney disease (CKD) and related complications, especially in elderly population. We attempted to verify the association between GFR and concurrent CKD complications and elucidate the temporal relationship between incident CKD and new CKD complications in a community-based prospective elderly cohort.

**Method:**

We analyzed the available data from 984 participants in the Korean Longitudinal Study on Health and Aging. Participants were categorized into 6 groups according to eGFR at baseline examination (≥90, 75–89, 60–74, 45–59, 30–44, and <30 ml/min/1.73 m^2^).

**Result:**

The mean age of study population was 76 ± 9.1 years and mean eGFR was 72.3 ± 17.0 ml/min/1.73 m^2^. Compared to eGFR group 1, the odds ratio (OR) for hypertension was 2.363 (95% CI, 1.299-4.298) in group 4, 5.191 (2.074-12.995) in group 5, and 13.675 (1.611-115.806) in group 6; for anemia, 7.842 (2.265-27.153) in group 5 and 13.019 (2.920-58.047) in group 6; for acidosis, 69.580 (6.770-715.147) in group 6; and for hyperkalemia, 19.177 (1.798-204.474) in group 6. Over a 5-year observational period, CKD developed in 34 (9.6%) among 354 participants with GFR ≥ 60 ml/min/1.73 m^2^ at basal examination. The estimated mean number of new complications according to analysis of co-variance was 0.52 (95% CI, 0.35–0.68) in subjects with incident CKD and 0.24 (0.19–0.29) in subjects without CKD (p = 0.002). Subjects with incident CKD had a 2.792-fold higher risk of developing new CKD complications. A GFR level of 52.4 ml/min/1.73 m^2^ (p = 0.032) predicted the development of a new CKD complication with a 90% sensitivity.

**Conclusion:**

In an elderly prospective cohort, CKD diagnosed by current criteria is related to an increase in the number of concurrent CKD complications and the development of new CKD complications.

## Introduction

Age is one of the most important risk factors for chronic kidney disease (CKD). In the United States, the prevalence of an estimated glomerular filtration rate (eGFR) of <60 ml/min/1.73 m^2^ increases from 0.9% to 51.2% with an increase in age from <60 years to ≥80 years [1]. However, it is debatable whether mildly decreased eGFR without definite evidence of renal damage, such as proteinuria or azotemia-related complications, should be considered an indicator of “disease” in the elderly population [2]. The reason for using an eGFR of 60 ml/min/1.73 m^2^ as a threshold value in the diagnoses of CKD is that eGFR < 60 ml/min/1.73 m^2^ is related to higher prevalence of CKD complications. In a prospective cohort study of 1,038 hospital inpatients, the eGFR thresholds used for detecting CKD complications were 50, 44, 40, 39, and 37 ml/min/1.73 m^2^ for hyperparathyroidism, anemia, acidosis, hyperkalemia, and hyperphosphatemia, respectively [3]. However, most of the study included relatively young patients, and thus its results may not be generalizable to the elderly population. In an examination of age-specific associations between eGFR and concurrent CKD complications by analysis of cross-sectional data collected from the National Health and Nutrition Examination Survey (NHANES) from 1988 to 1994 and from 1999 to 2006, Bowling et al. concluded that decreased eGFR is associated with a higher prevalence of CKD complications, regardless of age [4]. However, their use of a cross-sectional design did not allow for evaluation of the temporal association between decreased eGFR and CKD complications [4]. So, we constructed this study in order to verify the association between eGFR and concurrent CKD complications and elucidate the temporal relationship between incident CKD and new CKD complications in a community-based prospective elderly cohort. 

## Methods

### Design of KLoSHA

**Figure 1:**
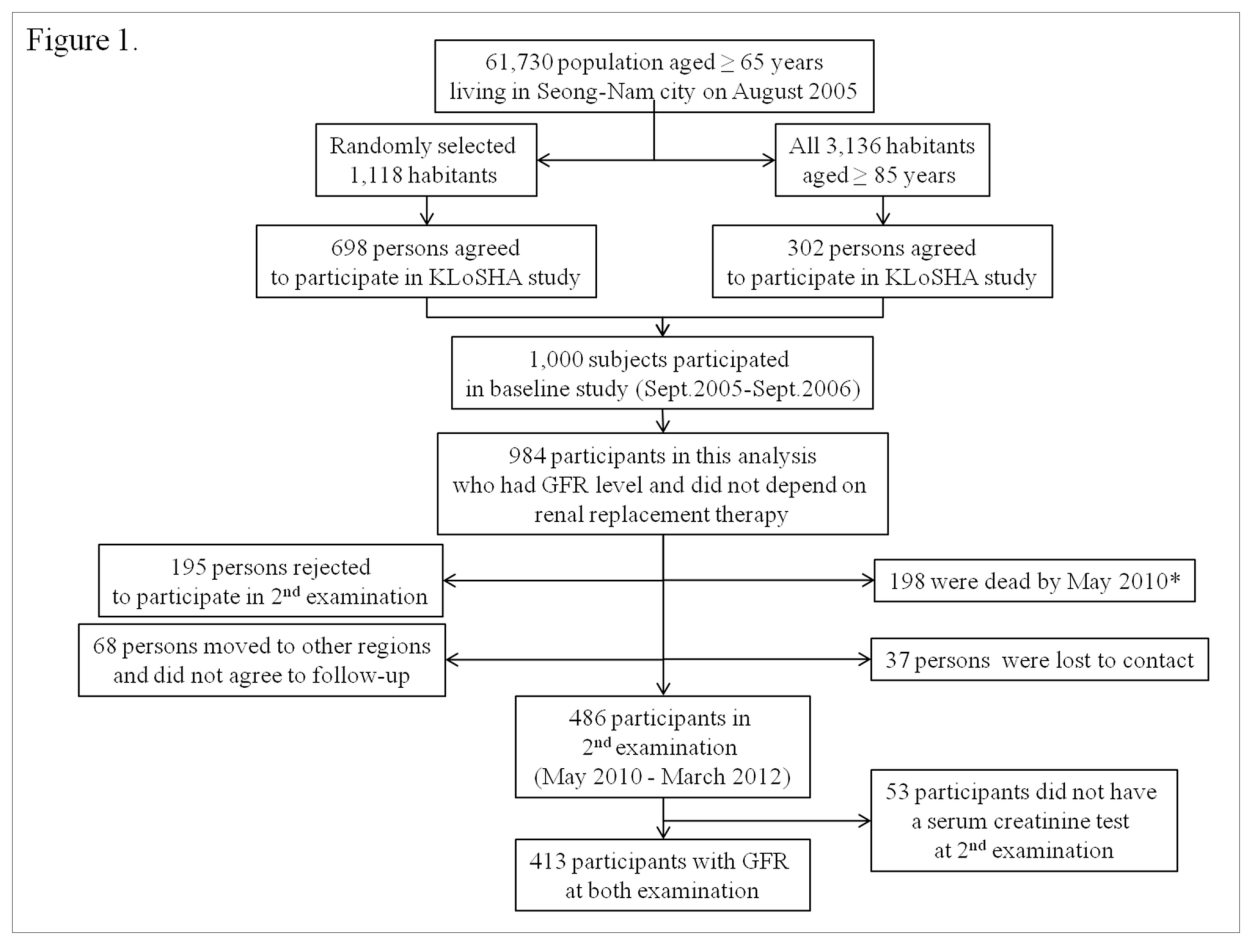
The participants in this study from the KLoSHA cohort.

This study was conducted as a part of the Korean Longitudinal Study on Health and Aging (KLoSHA), which included a randomly selected, community-based, elderly population [5]. The KLoSHA protocol was approved by the Institutional Review Board of Seoul National University Bundang Hospital (SNUBH) in 2005 and 2010 and accorded with the Declaration of Helsinki. The duration of the follow-up period was 59.4 ± 6.9 months. After obtaining written informed consent from all participants, the assessments were performed at SNUBH. Among the 1,000 original KLoSHA subjects, 984 subjects who were not dependent on renal replacement therapy and had a baseline value of glomerular filtration rate were selected for participation in this study (Figure 1). 

### Clinical characteristics

The data regarding all medications taken by the participants were collected. Diabetes mellitus (DM) and hypertension were defined as they had been elsewhere [5]. The serum creatinine level was measured by the alkaline picrate Jaffe kinetic method using an automatic analyzer (Toshiba 200FR; Tokyo, Japan). Serum creatinine levels were calibrated to an assay traceable on an isotope dilution mass spectrometry (IDMS) device (Roche Diagnostics). GFR was calculated using the CKD-epidemiology collaboration (CKD-EPI) equation [6]. The participants were categorized into 6 GFR groups: group 1, GFR ≥ 90 ml/min/1.73 m^2^; group 2, 75–89 ml/min/1.73 m^2^; group 3, 60–74 ml/min/1.73 m^2^; group 4, 45–59 ml/min/1.73 m^2^; group 5, 30–44 ml/min/1.73 m^2^; and group 6, <30 ml/min/1.73 m^2^. CKD was defined as a GFR < 60 ml/min/1.73 m^2^. Anemia was defined as a hemoglobin level < 12 g/dL in women and <13 g/dL in men [7]. Nutrient deficiencies were defined as follows: iron deficiency as the total iron-binding capacity (TIBC) saturation rate < 15% [8], ferritin deficiency as a ferritin level < 12 ng/ml [8], vitamin B12 deficiency as a B12 level < 200 pg/ml [9], and folate deficiency as a folate level < 2.6 ng/ml [9]. Hypertriglyceridemia was defined as serum triglyceride levels ≥ 150 mg/dL, hypoalbuminemia as serum albumin levels < 3.5 g/dL, hyperkalemia as serum potassium levels > 5.5 mEq/L, and acidosis as total CO_2_ < 22 mmol/L.

### Outcomes

The duration of the follow-up period was 59.4 ± 6.9 months. Complications related to CKD were defined as hypertension, hypertriglyceridemia, hypoalbuminemia, hyperkalemia, acidosis, and anemia. Incident CKD was diagnosed in patients who experienced GFR decrease from ≥60 ml/min/1.73 m^2^ at baseline examination to <60 ml/min/1.73 m^2^ at second examination and persistent CKD in patients who had GFR <60 ml/min/1.73 m^2^ at both baseline and second examination. Newly developed CKD complications were detected at second examination. 

### Statistical analyses

All analyses were performed using SPSS software (SPSS version 20.0, Chicago, IL). Data were presented as the mean ± SD values for continuous variables and as proportions for categorical variables. Differences in continuous variables were analyzed by one-way analysis of variance or Student *t*-test according to the number of subgroups, and differences in categorical variables were analyzed by Pearson’s chi-square or Fisher’s exact test. The levels of potassium, total CO_2_, and hemoglobin and the number of complications at baseline examination were estimated by analysis of co-variance (ANCOVA), adjusted by the factors related to each parameter. The risk factors for concurrent CKD complications, incident CKD, persistent CKD, and newly developed CKD complications during follow-up were identified by multiple logistic regression analysis. Formal testing for interactions between GFR groups and aging for each complication was conducted by comparing –2 log likelihood in regression models with and without the interaction term (GFR group*age in a 10-year unit). 

## Results

### Basal characteristics

The patient sample (N = 984) included 436 men (44.3%) and 548 women (55.7%) with a mean age of 76.0 ± 9.1 years and a mean estimated GFR of 72.3 ± 17.0 ml/min/1.73 m^2^. The number of participants in each GFR group was as follows: 150 (15.2%) in group 1 (GFR ≥ 90 ml/min/1.73 m^2^), 348 (35.4%) in group 2 (GFR 75–89 ml/min/1.73 m^2^) 249 (25.3%) in group 3 (GFR 60–74 ml/min/1.73 m^2^), 161 (16.4%) in group 4 (GFR 45–59 ml/min/1.73 m^2^), 59 (6.0%) in group 5 (GFR 30–44 ml/min/1.73 m^2^), and 17 (1.5%) in group 6 (GFR <30 ml/min/1.73 m^2^; Table 1). 

**Table 1:** Basal characteristics of elderly population at 1^st^ examination according to GFR.

	GFR group						p-value
	Group 1	Group 2	Group 3	Group 4	Group 5	Group 6	
Number	150	348	249	161	59	17	
Age (years)	69.2±5.2	74.6±8.3	76.2±8.3	81.9±9.8	82.7±8.0	83.0±7.0	<0.001
Gender (Male, %)	33.3	42.8	47.0	54.0	42.4	47.1	0.012
DBP (mmHg)	83±10	84±10	82±10	82±11	80±12	84±14	0.171
SBP (mmHg)	133±18	133±18	131±16	133±19	131±23	136±22	0.831
History of cancer (%)	8.8	5.5	6.9	10.0	5.2	5.9	0.476
Smoking (%)							0.001
Never	71.3	61.8	54.2	49.1	57.6	70.6	
Ex-smoker	16.7	25.9	33.3	39.1	39.0	23.5	
Current smoker	12.0	12.4	12.4	11.8	3.4	5.9	
DM (%)	24.0	23.6	24.5	22.4	27.1	29.4	0.971
Hypertension (%)	67.3	72.4	72.7	77.6	84.7	94.1	0.032
Glucose (mg/dL)	110±30	110±27	108±22	106±21	103±24	105±21	0.325
HbA1c (%)	6.1±1.0	6.0±0.8	6.0±0.8	6.0±0.7	6.0±0.6	6.0±1.2	0.932
Albumin (g/dL)	4.1±0.2	4.1±0.3	4.1±0.2	4.1±0.2	4.1±0.3	4.0±0.3	0.167
Cholesterol (mg/dL)	205±38	203±38	202±39	201±34	203±40	194±40	0.869
Triglyceride (mg/dL)	143±108	129±80	138±81	133±1	127±46	135±63	0.496
Creatinine (mg/dL)	0.65±0.11	0.79±0.13	0.94±0.13	1.12±0.15	1.41±0.23	2.49±1.50	<0.001
GFR (ml/min/1.73 m^2^)	94.1±5.0	82.2±4.9	68.0±4.1	54.4±4.1	39.7±3.8	24.2±7.0	<0.001
CRP (mg/dL)	0.21±0.56	0.24±0.65	0.22±0.49	0.24±0.52	0.61±1.71	0.14±0.22	0.003
TIBC saturation (%)	30.8±12.7	32.1±13.7	33.3±12.4	30.7±10.6	27.9±10.3	30.4±16.3	0.038
Ferritin (ng/mL)	107±151	121±116	119±98	125±104	128±131	147±115	0.672
Vitamin B12 (pg/ml)	629±322	667±927	639±347	687±995	617±343	979±849	0.521
Hemoglobin (g/dL)	13.7±1.4	13.8±1.5	13.9±1.4	13.6±1.5	12.7±1.5	12.4±1.2	<0.001
Proteinuria by dipstick (%)							<0.001
None	89.6	86.8	86.3	77.1	63.8	56.3	
trace	4.9	6.6	7.5	13.4	15.5	6.3	
1+ or more	5.6	6.6	6.2	9.5	20.7	37.6	
Hematuria (%)	7.6	10.5	11.7	10.2	10.3	25.0	0.394
Medication (%)							
ACEI or ARB	8.7	11.2	13.7	17.4	25.4	52.9	<0.001
Anti-platelet agent	11.3	17.8	24.1	23.6	28.8	47.1	0.001
Antidiabetic medication	10.7	10.3	12.9	13.7	15.3	23.5	0.488
Statin	5.3	7.2	8.4	10.6	10.2	5.9	0.584
No.of anti-HTN (%)	37.3	44	44.6	50.3	62.7	94.1	<0.001
1	26.0	30.5	26.9	29.8	32.2	41.2	
2	7.3	9.8	9.6	13.0	13.6	29.4	
3	3.3	3.4	6.8	5.0	13.6	5.9	
>3	0.7	0.3	1.2	2.4	3.4	17.6	

GFR: Calculated by CKD-EPI equation, , GFR groups; group 1: ≥ 90, group 2: 75-89, group 3: 60-74, group 4 : 45-59, group 5: 30-44, and group 6 : < 30 ml/min/1.73 m^2^, BMI: body mass index, CRP: c-reactive protein, TIBC: Total iron binding capacity, Proteinuria: measured by dipstick test, Hematuria: RBC ≥ 5/HPF, Diabetic medication: oral hypoglycemic agent, insulin, and/or others, No. of anti-HTN: number of anti-hypertensive medication

### Complications at baseline

**Figure 2:**
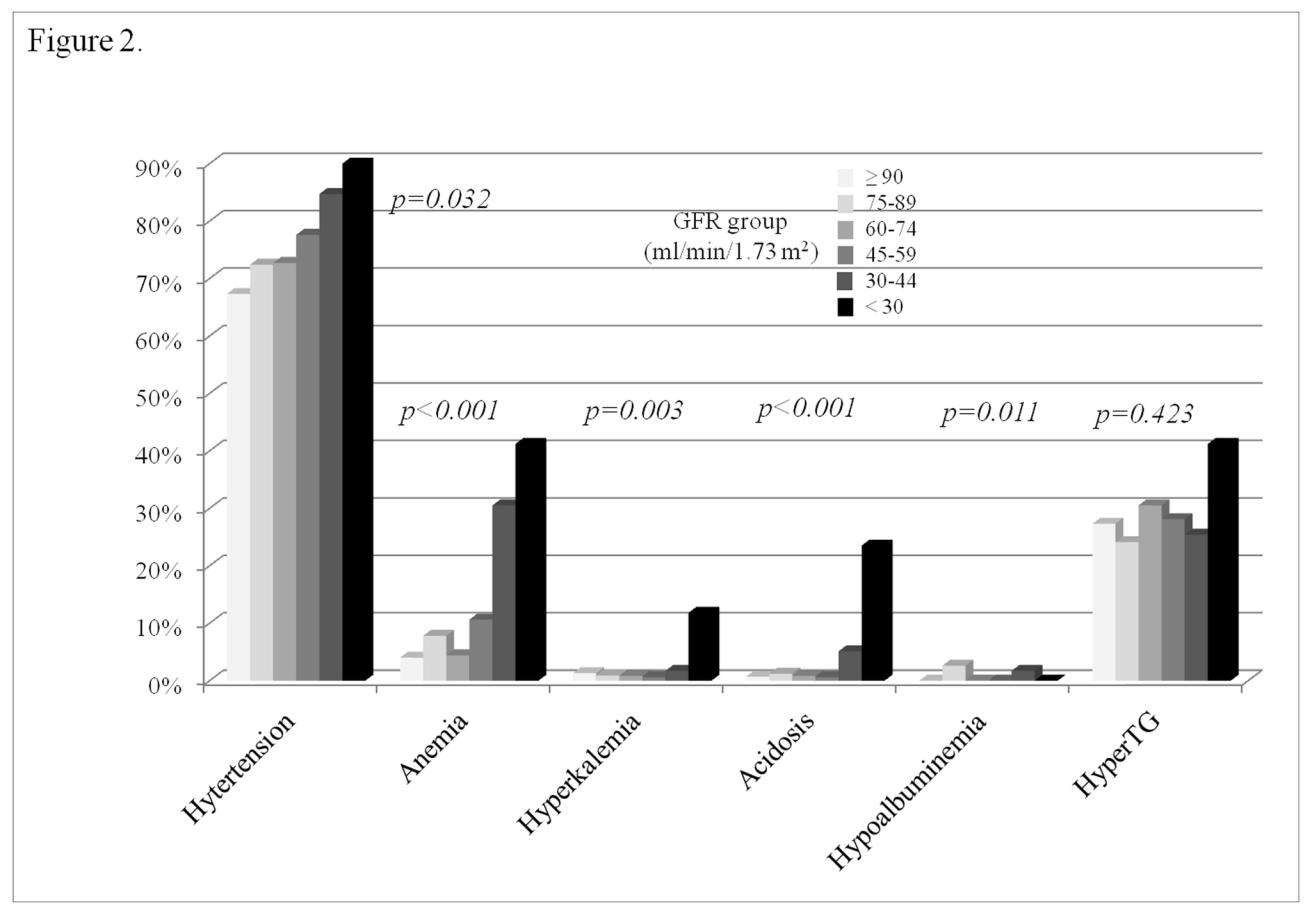
The prevalence of complications related to renal dysfunction. P-value calculated by Pearson’s Chi-square test. HyperTG: hypertriglyceridemia.

**Figure 3:**
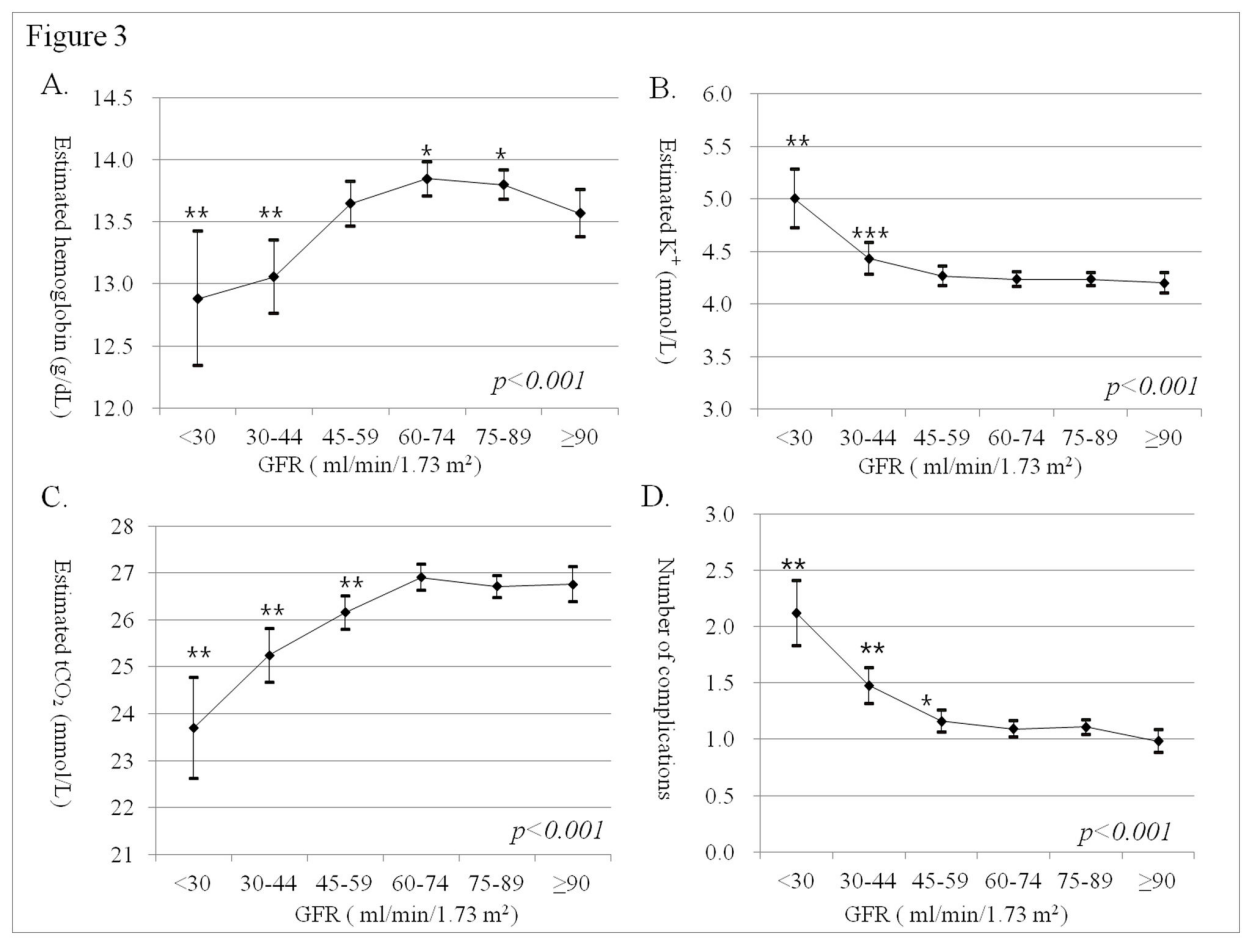
Estimated hemoglobin level (A), serum potassium level (B), total CO_2_ level (C), and number of complications (D) according to GFR group at basal examination by ANCOVA. *p < 0.05, different from GFR group 1; **p < 0.05, different from other GFR groups; ***p < 0.05, different from GFR groups 1, 2, and 3. The level of hemoglobin was adjusted by age; sex; SBP; DBP; number of anti-hypertensive medications; ferritin deficiency; presence of anti-HBV surface antibodies; levels of serum albumin, alanine aminotransferase, bilirubin, gamma-glutamyl transferase, cholesterol, triglyceride, CRP, and HbA1c; TIBC saturation rate; activated prothrombin time; and WBC count. The level of serum potassium was adjusted by age; sex; levels of serum bilirubin, thyroxin 4, and LDH; and TIBC saturation rate. The level of total CO_2_ was adjusted by age, sex, and presence of proteinuria ≥ 1+. The number of complications was adjusted by age; sex; SBP; DBP; levels of glucose, albumin, alkaline phosphatase, alanine aminotransferase, gamma-glutamyl transferase, cholesterol, HDL-cholesterol, triglycerides; TIBC saturation rate; WBC and platelet count; and ESR.

With decrease of GFR level, higher prevalence of hypertension, anemia, hyperkalemia, or acidosis were notified (Figure 2). The odds ratio (OR) for hypertension increased in GFR group 4, group 5, and group 6, for anemia, in GFR group 5 and group 6, for acidosis or hyperkalemia, in GFR group 6, compared to GFR group 1 (Table 2). There were mild positive interactions between GFR group and age for hypertension (p-interaction=0.046) but, not for acidosis, anemia, hypoalbuminemia, and hyperkalemia. The estimated hemoglobin was higher in GFR group 2 or group 3 than in group 1, although, that was lower in Group 5 or group 6 (Figure 3). The estimated potassium level in group 6 was lower than in other groups (p<0.001). The estimated total CO_2_ started to decrease from GFR 60 ml/min/1.73 m^2^ (p<0.001). The estimated number of complications adjusted with related factors was 0.98 (0.88-1.09) in GFR group 1, 1.11 (1.05-1.17) in group 2, 1.09 (1.02-1.17) in group 3, 1.16 (1.06-1.26) in group 4, 1.48 (1.32-1.64) in group 5, and 2.12 (1.83-2.41) in group 6 (p<0.001) (Figure 3). 

**Table 2:** The odds ratio for complications in GFR groups at basal examination.

	B	Wald	OR	95% CI for OR		p-value
Hypertension^1^		19.375				0.002
GFR group 1 vs 2	0.444	3.032	1.558	0.946	2.567	0.082
GFR group 1 vs 3	0.475	3.134	1.607	0.950	2.719	0.077
GFR group 1 vs 4	0.860	7.927	2.363	1.299	4.298	0.005
GFR group 1 vs 5	1.647	12.378	5.191	2.074	12.995	<0.001
GFR group 1 vs 6	2.614	5.745	13.657	1.611	115.806	0.017
Anemia^2^		31.997				<0.001
GFR group 1 vs 2	0.459	0.694	1.583	0.537	4.663	0.405
GFR group 1 vs 3	-0.155	0.063	0.856	0.255	2.872	0.801
GFR group 1 vs 4	0.919	2.250	2.508	0.754	8.336	0.134
GFR group 1 vs 5	2.059	10.562	7.842	2.265	27.153	0.001
GFR group 1 vs 6	2.566	11.324	13.019	2.920	58.047	0.001
Acidosis^3^		30.231				<0.001
GFR group 1 vs 2	0.419	0.130	1.521	0.156	14.806	0.718
GFR group 1 vs 3	0.379	0.095	1.461	0.131	16.342	0.758
GFR group 1 vs 4	0.219	0.024	1.245	0.077	20.255	0.878
GFR group 1 vs 5	2.203	3.543	9.054	0.913	89.779	0.060
GFR group 1 vs 6	4.242	12.736	69.580	6.770	715.147	<0.001
Hyperkalemia^4^		12.179				0.032
GFR group 1 vs 2	0.093	0.007	1.098	0.130	9.262	0.932
GFR group 1 vs 3	-0.052	0.002	0.950	0.098	9.218	0.965
GFR group 1 vs 4	-0.216	0.025	0.805	0.055	11.745	0.874
GFR group 1 vs 5	0.789	0.330	2.200	0.149	32.396	0.566
GFR group 1 vs 6	2.954	5.983	19.177	1.798	204.474	0.014
CKD complication^5^		14.801				0.005
GFR group 1 vs 2	0.496	3.119	1.643	0.947	2.850	0.077
GFR group 1 vs 3	0.634	4.363	1.884	1.040	3.414	0.037
GFR group 1 vs 4	0.635	3.486	1.887	0.969	3.676	0.062
GFR group 1 vs 5 & 6	2.285	13.400	9.828	2.891	33.407	<0.001

Hypertension^1^: Odds ratio of GFR group for hypertension, adjusted with age, gender, SBP, DBP, DM, and levels of serum albumin, alanine aminotransferase, cholesterol, triglyceride, HbA1c, TIBC, WBC, and platelet.

Anemia^2^: Odds ratio of GFR group for anemia, adjusted with age, gender, SBP, DBP, number of anti-hypertensive medication, deficiency of ferritin, presence of anti-HBV surface antibody, and levels of serum albumin, alanine aminotransferase, bilirubin, Gamma-glutamyl transferase, cholesterol, triglyceride, CRP, HbA1c, TIBC saturation rate, activated prothrombin time, and WBC.

Acidosis^3^: Odds ratio of GFR group for acidosis, adjusted with age, gender, and presence of proteinuria ≥1+.

Hyperkalemia^4^: Odds ratio of GFR group for hyperkalemia, adjusted with age, gender, serum bilirubin, thyroxin 4, TIBC,and LDH

CKD complication^5^ : Odds ratio of GFR group, in which group 5 and 6 were combined because of lack of subjects without complication in group 6, for presence of CKD complications, adjusted with age, gender, SBP, DBP, glucose, albumin, alkaline phosphatase, alanine aminotransferase, gamma-glutamyl transferase, cholesterol, HDL-cholesterol, triglyceride, TIBC, WBC, platelet, ESR.

The factor of GFR group was not related to hypoalbuminemia and hypertriglyceridemia by mulitple logistic regression adjusted with other risk factors. CKD complications included anemia, acidosis, hyperkalemia, hypoalbuminemia, hypertriglyceridemia, and hypertension.

GFR groups; group 1: ≥ 90, group 2: 75-89, group 3: 60-74, group 4 : 45-59, group 5: 30-44, and group 6 : < 30 ml/min/1.73 m^2^

### Incident and persistent CKD

Among 354 participants who had a GFR ≥ 60 ml/min/1.73 m^2^ at basal examination, 34 developed CKD (9.6%) during a 5-year observational period. The risk of incident CKD was found to be 5.896-fold higher in group 3 as compared to group 1 (p = 0.006). Each 10-mmHg increase in SBP was found to increase risk of incident CKD by 1.331-fold (95% CI: 1.087–1.629) (Table 3). Compared to participants without incident CKD, the prevalence of anemia (9/36 vs. 25/318 participants; p = 0.001) and number of CKD complications (1.58 ± 0.61 vs. 1.13 ± 0.72 complications; p = 0.001) were found to be higher in participants with incident CKD. Among 59 subjects with a GFR < 60 ml/min/1.73 m^2^ at basal examination, GFR increased to ≥60 ml/min/1.73 m^2^ in 20 (33.9%). Although 46.3% (19/41) of participants with GFR of 45–59 ml/min/1.73 m^2^ at basal examination had a GFR ≥60 ml/min/1.73 m^2^ at second examination, only 5.6% (1/18) who had a GFR <45 ml/min/1.73 m^2^ at baseline had the above mentioned GFR at the second examination (p = 0.002). The number of CKD complications at the second examination was higher in participants with persistent CKD than in participants with GFR who experienced a GFR increase to over 60 mL/min/1.73 m^2^ (1.79 ± 0.96 vs. 1.00 ± 0.65; p = 0.001). 

**Table 3:** The risk factors for incident CKD or persistent CKD among subjects with follow-up examination.

		B	RR	95% CI for RR		p-value
Incident CKD*	GFR (ml/min/1.73 m^2^)					0.010
	≥ 90 vs 74-89	0.995	2.705	0.730	10.028	0.137
	≥ 90 vs 60-74	1.781	5.934	1.671	21.075	0.006
	SBP (mmHg)					0.007
	<130 vs 130-149	0.526	1.692	0.659	4.349	0.275
	<130 vs ≥150	1.447	4.251	1.661	10.883	0.003
Persistent CKD**	GFR < 45 ml/min/1.73 m^2^	1.896	6.661	0.645	68.805	0.111
	Age (year)	0.151	1.163	1.047	1.292	0.005
	DM (presence)	3.579	35.854	2.223	578.167	0.012
	Bilirubin (mg/dL)	3.994	0.018	0.001	0.384	0.010

^*^ Incident CKD: The relative risk for GFR <60 ml/min/1.73 m^2^ at 2^nd^ examination was analyzed among participants with GFR ≥60 ml/min/1.73 m^2^ at basal examination, which was estimated with adjustment by age, gender, DM, hypertension, SBP, presence of proteinuria ≥ 1+, and GFR (≥ 90, 75-89, and 60-74 ml/min/1.73 m^2^) at baseline examination.

^**^ Persistent CKD: The relative risk for GFR <60 ml/min/1.73 m^2^ at 2^nd^ examination was analyzed among participants with GFR <60 ml/min/1.73 m^2^ at basal examination, which was estimated with adjustment by age, gender, DM, hypertension, presence of proteinuria ≥ 1+, bilirubin, hemoglobin, and GFR (≥ 45 and < 45 ml/min/1.73 m^2^) at baseline examination.

### Newly developed CKD complications

Among the 354 subjects who had a GFR ≥ 60 ml/min/1.73 m^2^ at basal examination, 90 (25.4%) developed at least one new complication during the follow-up period. These included 40 (11.3%) subjects who developed incident hypertriglyceridemia; 27 (7.6%), incident anemia; 17 (4.8%), incident hypertension; 5 (1.4%), incident hyperkalemia; 3 (0.8%), incident acidosis; and 3 (0.8%), incident hypoalbuminemia. Simple logistic regression analysis indicated that incident CKD increases the risk of developing anemia (relative risk [RR]: 4.874, 95% CI: 1.946–12.208, p = 0.001) and hyperkalemia (RR: 6.604, 95% CI: 1.064–40.995, p = 0.043), and multiple regression analysis indicated that incident CKD increases the risk of developing new complications by 2.792-fold (95% CI: 1.308–5.964). ANCOVA adjusted by related factors for development of new CKD complications indicated that subjects with incident CKD developed 0.52 (95% CI: 0.35–0.68) new complications, while subjects without incident CKD developed 0.24 (95% CI: 0.19–0.29; p = 0.002) new complications. Among participants with a GFR ≥ 60 ml/min/1.73 m^2^ at baseline examination, a threshold GFR of 52.4 ml/min/1.73 m^2^ at second examination was found to predict development of any CKD complication with 90% sensitivity (p = 0.032 by ROC analysis).

## Discussion

In the cohort examined in this study, the concurrent prevalence of hypertension increased at a threshold GFR of <60 ml/min/1.73 m^2^, anemia at GFR < 45 ml/min/1.73 m^2^, and both acidosis and hyperkalemia at GFR < 30 ml/min/1.73 m^2^. The rate of incident CKD, as defined by the criterion value of GFR of 60 ml/min/1.73 m^2^, was 9.6% over the 5-year study period. The rate of the incident CKD was found to increase the incidence of anemia and hyperkalemia and the total number of newly developed complications, while improvement in GFR was found to result in improvement of the severity of complications.

Several reports have suggested that a decrease in GFR in older subjects increases the risk for metabolic complications associated with CKD. However, these reports were limited in their analysis of the temporal associations between complications and decrease in GFR because of cross-sectional or retrospective study design [4,10]. The KLoSHA offered several advantages in this respect, being based on analysis of data representative of a city population collected from non-institutionalized inhabitants selected by random sampling. A prospective study, the KLoSHA, provides higher-quality evidence and allows for analysis of the temporal relationship between incident CKD and complications. In addition, the KLoSHA measured serum creatinine levels in a single institution throughout the study period.

The results of this study confirm the existence of a relationship between the concurrent CKD complications and a decrease in GFR without any interactions between GFR and the aging process, indicating that a decrease in GFR attributable to the aging process may not be responsible for these complications. When we adopt the criteria for GFR group (≥60, 45-59, <45 ml/min/1.73 m^2^), the higher prevalence of anemia, acidosis, hyperkalemia, hypertension, and CKD complication as a whole were consistently present at GFR levels <45 ml/min/1.73 m^2^ as the results of elderly subjects from NHANES data [4]. Overall number of concurrent complications related to CKD started to increase at GFR <60 ml/min/1.73 m^2^ which was comparable to the Gubbio study in Italy [11]. 

Although the changes in total CO_2_ and potassium levels and number of complications observed with a decrease in GFR were linear, the changes observed in the estimated hemoglobin level were non-linear. Specifically, the results of ANCOVA revealed that estimated hemoglobin levels increased with a decrease in GFR, beginning at an initial GFR of 90 ml/min/1.73 m^2^ until it reached 45 ml/min/1.73 m^2^ and then began to decrease with a continued decrease in GFR, a pattern that we had observed in a younger population [12]. Such a phenomenon could not be related to iron, vitamin B12, or folate deficiency because the prevalence of deficiency did not differ according to the GFR decline. A pattern of changes in hemoglobin level was also observed in subjects without nutrient deficiency (Figure S1), which might correlate with the findings of early research into erythropoietin and creatinine clearance [13]. Specifically, hematocrit and erythropoietin levels were found to slightly increase with a decrease in creatinine clearance from 90 to approximately 45 ml/min, and the erythropoietin level of patients with a high BMI and DM was found to be 22–55% higher than that of patients without these disorders [14]. These findings suggest that a slight increase in the hemoglobin level, associated with a minor decrease in GFR beginning at a GFR of 90 up till 60 ml/min/1.73 m^2^ and/or cardio-renal risk factors, may result from an increase in renal hypoxia aggravated by pre-existing conditions [15]. 

As in a previous study [4], hypoalbuminemia was not found to be related to GFR in the present study. Hypertriglyceridemia in end-stage renal disease is accompanied by impaired clearance of triglyceride-rich lipoproteins caused by dysregulation of lipoprotein lipase, hepatic lipase, or the very low-density lipoprotein receptor [16]. However, as the prevalence of hypertriglyceridemia depends on the severity of renal dysfunction, it tends not to increase in cases of mild-to-moderate CKD [17]. Another factor in the prevalence of hypertriglyceridemia related to CKD is the extent of proteinuria. Although significant proteinuria results in a significant increase in triglyceride synthesis [18], the prevalence of significant proteinuria (≥ 2+) was minimal (2.8%) in the population examined in the present study. 

GFR and SBP were reported as the most important risk factors of incident CKD in previous reports [15,19,20]. In non-diabetic elderly subjects of the Cardiovascular Health Study Cohort, the OR for increase of serum creatinine ≥0.3 mg/dL was found to be 1.16 for every 10 mmHg increase in SBP [19]. In community-based cohort studies [19,21], baseline renal function was found to be an independent factor for renal dysfunction or incident CKD. In the present study, subjects with incident CKD were found to have a higher number of concurrent complications and newly developed complications than subjects without CKD at second examination, a finding that provides good evidence of the existence of a positive temporal relationship between CKD and the number and frequency of complications. Using the criterion of GFR of 60 ml/min/1.73 m^2^ to define CKD, the incidence of anemia and hyperkalemia was found to have significantly increased at the second examination. Moreover, a significant difference in the number of complications was found between subjects with persistent CKD and with improved CKD, whose GFR had increased to ≥60 ml/min/1.73 m^2^ from <60 ml/min/1.73 m^2^. Although it is debatable whether “improved CKD” can be defined by an improvement in serum creatinine levels at the second examination, suggested imprecision of creatinine-based GFR compared to cystatin-based GFR within a GFR range of 45 to 59 ml/min/1.73 m^2^ for predicting outcomes [22], and lack of validation of CKD-EPI equation in older population, the significant decrease of number of complications according to increase of GFR from <60 ml/min/1.73 m^2^ to ≥60 ml/min/1.73 m^2^ should be notified. 

There are several limitations to be considered in the interpretation of the results of this study. First, the study attempted to follow-up the participants for 5 years, but 30.5% of the initial sample could not be followed up at the second examination. Second, the CKD-EPI equation has not been fully validated for use with elderly Koreans, although a study of 131 CKD patients and healthy volunteers found that the ethnic coefficient of the MDRD equation using IDMS-traceable creatinine value was close to 1 [23]. Third, serum creatinine was measured once at each examination, which could provoke a bias for classification with estimated GFR. Fourth, in the analysis of anemia, causes other than renal dysfunction could have been responsible for it, although the analysis was adjusted for iron, vitamin B12, and folate deficiency. Finally, as this study examined an urban population, the extent to which the results can be generalized to a nationwide population or all Asians is debatable.

In conclusion, measurement of GFR and use of the recommended criteria for diagnosis of CKD were found useful in the predicting concurrent as well as incident complications related to renal dysfunction in an elderly cohort, as was found in younger populations. Clinicians should attempt to identify complications related to CKD in older patients experiencing a mild-to-moderate decrease in GFR.

## Supporting Information

Figure S1 **Hemoglobin level according to GFR group in subjects without iron, vitamin B12, or folate deficiency.** The hemoglobin level was estimated by ANCOVA adjusted by factors related to hemoglobin level. Among 892 subjects without nutrient deficiency, hemoglobin level in subjects with a GFR > 90 ml/min/1.73 m^2^ was lower than that in subjects with a GFR of 60–74 ml/min/1.73 m^2^ (^*^p = 0.038), but higher than that in subjects with a GFR of 30–44 ml/min/1.73 m^2^ (^**^p = 0.001) and those with a GFR < 30 ml/min/1.73 m^2^ (^***^p = 0.015).
(TIF)
